# Humerus Diaphysis Fracture in a Newborn during Vaginal Breech Delivery

**DOI:** 10.1155/2015/489108

**Published:** 2015-12-03

**Authors:** Baris Kaya, Korkut Daglar, Ayse Kirbas, Abdullah Tüten

**Affiliations:** ^1^Gaziantep Maternity Hospital, Gaziantep, Turkey; ^2^Department of Perinatology, Zekai Tahir Burak Women's Health Education and Research Hospital, 06680 Ankara, Turkey

## Abstract

While most obstetricians are familiar with fracture of the clavicle in newborns during birth, an unlucky minority of obstetricians has encountered long-bone fractures in newborns as well. This complication is traumatic not only for the neonate, but also for the family and the obstetrician; it is also difficult to explain. Fortunately, the long-term prognosis for fracture of the long bones is excellent. Both vaginal and cesarean breech deliveries and maneuvers can be responsible for birth traumas, including long-bone fractures. This case report presents a newborn with breech presentation delivered vaginally that resulted in humerus diaphysis fracture.

## 1. Introduction

Most obstetricians are familiar with fracture of the clavicle in newborns during birth; however, an unlucky few have encountered long-bone fractures in newborns as well. These fractures are traumatic not only for the neonate, but also for the family and the obstetrician; they are also difficult to explain. Although it has been reported that cesarean delivery may reduce the incidence of long-bone fractures, they still occur [1, 2]. Breech delivery, by either vaginal or cesarean route, remains the most common independent factor for long-bone fractures [1–3].

There is limited data regarding vaginal breech delivery after cesarean section [2–4]. To the best of our knowledge, there have been no reports of humerus or any other long-bone fracture after vaginal breech delivery with a scarred uterus. Herein, we describe a case of humerus diaphysis fracture in a newborn.

## 2. Case Presentation

A 35-year-old woman, gravida 3, para 2, was admitted to our hospital in active labor with a frank breech presentation in the 39th week. She had delivered her first baby vaginally four years earlier and her second baby by cesarean section due to fetal distress three years earlier. This time, she requested a vaginal delivery, and written informed consent was obtained. Her labor progressed spontaneously. Once the scapula was visible, the obstetrician rotated the infant 90° and gently swept the anterior arm by pressing on the inner aspect of the elbow, but the maneuver failed. The obstetrician then rotated the infant 180° in the reverse direction and swept the right arm out of the vagina; however, the left arm was still in the uterus. Despite attempting all types of maneuvers, the left arm could not be rescued. The pulsation rate of the umbilical cord was dropping, so the staff had to apply traction forcefully to the left arm to rescue it. After the left humerus broke with an audible “crack,” the head of the baby quickly delivered. The male baby's weight was 3500 g, and his one- and five-minute Apgar scores were 5 and 8, respectively.

**Figure 1:**
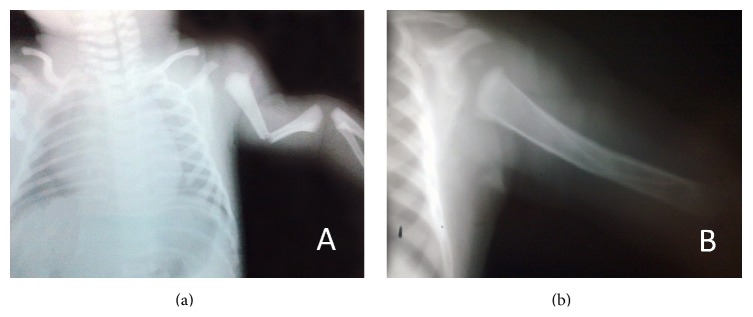
(a) Radiograph of fractured humerus prior to reduction and immobilization. (b) After 2 months.

The baby's broken arm (Figure 1(a)) was fixed to his body with a bandage and an orthopedic specialist was consulted. With immobilization, the baby's arm healed completely, without any deformity (Figure 1(b)).

## 3. Discussion

Forced obstetric maneuvers have been reported as a risk of soft-tissue injury, long-bone fractures, and related neonatal complications. Historically, long-bone fractures have been attributed to breech maneuvers during vaginal delivery; however, because cesarean deliveries are becoming more popular and include breech maneuvers, the incidence of long-bone fractures may be on the increase. Abdominal and vaginal delivery maneuvers are similar in breech presentation [4, 5]; while cesarean section avoids the risk of head entrapment, long-bone trauma can still occur [4–7].

Attempting a vaginal breech delivery after cesarean section is a challenging undertaking, and the rates of vaginal birth after cesarean section have been declining in recent years [4]. Nonetheless, with careful selection of patients and strict management of labor, vaginal birth does not seem to increase maternal or neonatal morbidity or mortality in this situation [6–8].

The incidence rate of long-bone injuries has been reported as 0.23–0.67 per 1,000 live births [9, 10]. To the best of our knowledge, there is only one case report in the literature of humerus fracture in a neonate related to breech vaginal delivery [3], and a few others have been reported in long-bone fracture series of newborns related to vaginal delivery [5].

In a series of seven neonates who sustained a birth-related fracture of the humerus, three cases were breech presentation, only one of which was due to vaginal breech delivery. Surprisingly, a significant number of fractures occurred in babies born via cesarean section unrelated to the presentation, which is considered to be safer than vaginal delivery [10]. Similarly, in their retrospective long-bone fracture series, Basha et al. concluded that emergency cesarean delivery carries a higher risk of long-bone fracture than vaginal delivery [5].

Forced breech maneuvers in both vaginal and cesarean routes of delivery, as well as emergency cesarean section, can cause fetal injuries, including long-bone fractures. Fortunately, humerus fractures heal with simple immobilization, without long-term deformity [11].

In conclusion, long-bone fractures are still one of the most feared complications of birth. It should be emphasized that cesarean section does not eliminate the possibility of long-bone fractures. It is important to inform patients scheduled for delivery due to breech presentation that long-bone fractures may occur due to the maneuvers performed during delivery. More importantly, it should also be emphasized that the risk cannot be eliminated completely, even if a cesarean section is performed. If the delivery is difficult, the newborn might experience trauma. Early detection of such traumas, including long-bone fractures, is very important; thus, a thorough examination and assessment are essential in the early period after the birth. A higher index of suspicion would help with early detection and treatment.
